# Clinical Consequences of Immune Response to CT Upper Genital Tract Infection in Women

**DOI:** 10.1155/S1064744996000361

**Published:** 1996

**Authors:** J. Henry-Suchet, M. Askienazy-Elbhar, J. Orfila

**Affiliations:** 1 Family Planning Centre Hospital Saint Louis 10 rue Marignan Paris 75008 France; 2 Magenta Laboratory Laboratory of Bacteriology and Immunology Paris France; 3 University hospital Amiens France

## Abstract

*C. TRACHOMATIS* (CT) infections of the upper genital tract in women are either acute, sub acute or chronic. CT infection has a tendency to be chronic, latent and persistent as a consequence of the host immune reaction to CT major outer membrane protein, 57 Kd heat shock protein and lipopolysaccharide. Chlamydial persistence can be induced as a result of inflammatory and/or immune regulated cytokines, Interferon γ depletion of tryptophan causes a stress response involving development of abnormal forms with increased levels of stress response proteins which maintain host immune responses with continuous fibrin exudate.

The main clinical consequences are acute and chronic pelvic inflammatory disease, with infertility, ectopic pregnancy and, less frequently, chronic pelvic pain as late sequelae.

PID, when acute, is marked by bilateral pelvic pain, plus other infectious signs in typical cases: fever, leucorrhea, red and purulent cervix. In 50% cases, infectious signs are slight or absent or there is an atypical clinical situation. Laparoscopy is the key for diagnosis. It allows the surgeon to have a direct look at the pelvic organs and perform microbiologic and histologic sampling. In severe cases, laparoscopy allows the surgeon to aspirate the purulent discharge and successfully treat pelvic abscesses.

Chronic PID usually is clinically silent. It is in most cases discovered some years after the onset of CT infection, in women operated on for tubal infertility or ectopic pregnancy. Further studies, to evaluate treatments efficiency in chronic cases and factors leading to ectopic pregnancy or to recurrence, are indicated.


Infectious Diseases in Obstetrics and Gynecology 4:171–175 (1996)
© 1996 Wiley-Liss, Inc.

### Clinical Consequences of Immune Response to CT Upper Genital Tract Infection in Women

J. Henry-Suchet,[1] M. Askienazy-Elbhar,[2] and J.Orfila[3]
[1]*Family Planning Centre, Hospital Saint Louis, Paris,* [2]*Magenta Laboratory, Paris, Laboratory of Bacteriology and Immunology,* [3]*University hospital, Amiens*

### Abstract

*C. TRACHOMATIS* (CT) infections of the upper genital tract in women are either acute, sub acute or chronic. CT infection has a tendency to be chronic, latent and persistent as a consequence of the host immune reaction to CT major outer membrane protein, 57 Kd heat shock protein and lipopolysaccharide. Chlamydial persistence can be induced as a result of inflammatory and/or immune regulated cytokines, Interferon γ depletion of tryptophan causes a stress response involving development of abnormal forms with increased levels of stress response proteins which maintain host immune responses with continuous fibrin exudate.

The main clinical consequences are acute and chronic pelvic inflammatory disease, with infertility, ectopic pregnancy and, less frequently, chronic pelvic pain as late sequelae.

PID, when acute, is marked by bilateral pelvic pain, plus other infectious signs in typical cases: fever, leucorrhea, red and purulent cervix. In 50% cases, infectious signs are slight or absent or there is an atypical clinical situation. Laparoscopy is the key for diagnosis. It allows the surgeon to have a direct look at the pelvic organs and perform microbiologic and histologic sampling. In severe cases, laparoscopy allows the surgeon to aspirate the purulent discharge and successfully treat pelvic abscesses.

Chronic PID usually is clinically silent. It is in most cases discovered some years after the onset of CT infection, in women operated on for tubal infertility or ectopic pregnancy. Further studies, to evaluate treatments efficiency in chronic cases and factors leading to ectopic pregnancy or to recurrence, are indicated. © 1996 Wiley-Liss, Inc.

Key words
*C. trachomatis; immune response; genital infection; women; pelvic inflammatory disease*

C. *trachomatis* (CT) serotypes D-K infections of the upper genital tract in women are either acute, sub acute or chronic. Due to the ascent of the bacteria from the cervix to the endometrium and Fallopian tubes, the main clinical consequences are acute and chronic pelvic inflammatory disease (PID), with infertility, ectopic pregnancy and, less frequently, chronic pelvic pain as late sequelae.[1,2]

CT infection is involved in 60% of acute salpingitis and ectopic pregnancies[3] and 80% of tubal infertility cases.[2,4]

CT infection has a tendency to be chronic, latent and persistent. This immunopathogenesis is a consequence of the host immune reaction to CT.[5] This obligate intracellular organism, with a slow reproductive cycle, generates an inflammatory host cascade due to its major outer membrane protein (MOMP), 57 Kd heat shock protein, which is a member of the family of 60 kD heat shock proteins (hsp 60) and lipopolysaccharide (LPS).[6] Host immunity to CT hsp60 is associated with upper and not to low genital tract infection (cervix, vagina), as shown by Witkin.[7] This response is both protective and pathological. MOMP is a likely protection-mediating antigen and a good candidate for vaccine, but this immune protection is serotype-specific and

Address correspondence to J. Henry-Suchet, M.D. Gynecologue Accoucheur, chargée de cours Université Hopital St Louis Paris, 10 rue Marignan 75008 Paris France.

Received September 15, 1996
Accepted October 1, 1996

Article of short duration.[8] Hsp 60 is probably the pathogenesis-inducing antigen.[9] Among the immune cascade multiple reactions of primary importance are: 1) the T lymphocyte's and macrophage's production of cytokines: IL4, IL6, Tumor Necrosis Factor (TNF), Interferon gamma (IFN$\gamma$) and 2) the genital and peritoneal tissues production of fibrin exudate. In vitro studies have shown IFN$\gamma$ able to block the reproductive cycle of CT from elementary to reticulate bodies, probably by depleting tryptophan. The reproductive cycle starts again if this amino acid is added to the culture.[10] A local immune response (T cells, IFN$\gamma$ . . . ) has been found in endometrial biopsies of women with tubal infertility or with a past history of chlamydial infection.

In some cases, the inflammatory cascade is of benefit, generating clinical signs which lead to diagnosis and, with antibiotic help, obtaining the organism destruction and host recovery.

In other cases, CT infection either turns from acute to chronic, or is primarily chronic. Chlamydial persistence can be induced as a result of inflammatory and/or immune regulated cytokines,[10] IFN$\gamma$ depletion of tryptophan causes a stress response involving development of abnormal forms with increased levels of stress response proteins. These altered CT antigens persist in the tissues and more particularly in tubal submucosa,[11,12] their presence continuing to maintain the immune host reaction. Furthermore, the CT membrane stress proteins, more particularly hsp 60, generate a host delayed immune hypersensitivity and maintain susceptibility to the inflammatory reaction and to reactivation by other antigens with amino acid homology to chlamydial hsp:s, such as E. coli GroEJ, GroEL and DnaK-like proteins.[13] This slow inflammatory process has as a consequence a continuous fibrin production. Tubal and pelvic damages are mainly due to fibroid exudate, either liquid in the cul-de-sac of Douglas, or solid, creating light or dense and thick adhesions glueing together ovaries and tubes and both to parietal peritoneum, uterus or bowels, glueing together tubal fimbria fringes and causing various degrees of tubal obstruction.[14] In other cases, the immune reaction remains very slight, without tubal occlusion, but mucosa ciliae are altered with defects in oocyte transport through the oviduct and ectopic pregnancies or, possibly, early spontaneous abortions as consequences.

The reasons why inflammatory responses lead to either acute, sub acute or chronic conditions is unclear to date. This can depend on the specificity of CT subgroups, or, more probably, on the coincidence between 1) number and frequency of repeated CT infections and 2) genetic variability of the host immune responses. While DHS reactions are relatively common, the potential to develop an auto immune tubal inflammatory response is possible only in women genetically determined to recognize the conserved epitopes of the hsp:s.[13] The true nature of CT chronic disease pathogenesis possibly involves repeated infection superimposed upon persistent infection which would allow for heightened immune reactivity to conserved antigens, irrespective of the infective serovar.

Clinical aspects of immune responses to CT infection of the upper genital tracts are well known.[15] CT endometritis is mostly infra clinical or leads to a slight bleeding. PID, when acute, is marked by bilateral pelvic pain, plus other infectious signs in typical cases: fever, leucorrhea, red and purulent cervix. In one case out of two, however, infectious signs are slight or absent or there is an atypical clinical situation: unilateral pain, adnexial mass, or intense perihepatic pain as part of a Fitz-Hugh-Curtis syndrome.[16] Fortunately, all practicians know now that they have to "think PID" when a young woman complains of pelvic pain, even if it is a discreet or atypical one.

### Diagnosis

The keys for diagnosis are: 1) biology and 2) laparoscopy

### *Biologic infectious signs*

Lymphocytosis, accelerated ESR and/or CRP are found in 50-75% of PID; their absence, however, does not contraindicate the diagnosis. STD detection in the lower genital tract and endometrium is therefore mandatory, more particularly *N. gonorrheae,* CT and mycoplasmas. CT serology on sera taken at a 3 week interval is a useful diagnosis tool.[17] According to our experience,[18] antiCT IgG usually is strongly positive and the titer is stable in 85% of cases, but it can be negative on first serum in 5% and evolutive on second serum in 10%. IgA is positive in 85% and evolutive in 30%, IgM is positive in 80% of cases in our published series, antichlamydial IgM antibody determination by immunofluorescence using *C. trachomatis*-infected cells being easier to read and yielding a higher incidence of positives than does immunofluorescence on purified elementary bodies. With the last method, IgM is occasionally positive. Anti CT hsp 60 antibody has been recently shown as a good marker of CT salpingitis, related to measures of tubal inflammation, tubal occlusion and/or adhesions at laparoscopy.[19]

### *Laparoscopy*

Laparoscopy is the gold standard for diagnosis. However, being an invasive and costly method, it is indicated mostly in atypical cases, severe cases and cases without improvement after some days of antibiotic treatment. Medical treatment of typical cases with 3 or more clinical and/or biological signs is recommended[20] and the effectiveness of this treatment can be considered as a complementary diagnosis test.[15]

Laparoscopy, first proposed by Jacobson and Westrom,[21] allows the surgeon to have a direct look at the pelvic organs and perform microbiologic and histologic intrapelvic sampling. In most cases, lesions are obvious: red and swollen tubes, purulent or thick and yellow peritoneal discharge, red inflammatory periadnexial adhesions. A Fitz-Hugh-Curtis syndrome with its perihepatic adhesions sometimes is associated with CT salpingitis, even in patients without hepatic pain. In severe cases, laparoscopy allows the surgeon to aspirate the purulent discharge and, together with a strong and polyvalent antibiotic therapy, successfully treat pelvic abscesses.[22] In contrast, laparoscopic observation may find a normal pelvis in recent or mild cases: in such cases it is recommended to do peritoneal and tubal micro biopsies and wait for histologic results before eliminating a PID diagnosis. Multiple laparoscopic sampling shows usually CT alone or associated with other agents in 50-60% of PID:s.[20,23]

Evolution under polyvalent antibiotic treatment has to be followed for clinical and biological signs, noticeably ESR and CRP decrease. The objective in young women will be to evaluate tubal sequelae. The chances for tubal infertility have been estimated at 17% after one salpingitis, 30% after two and 60% after three or more.[24] A recent Swedish study on a cohort of women hospitalised for chlamydial confirmed acute PID, shows, despite accurate treatment, a 5% tubal infertility risk and a 5% ectopic pregnancy risk.[25]

### Chronic PID

Chronic PID usually is silent, leading to little or no clinical pelvic pain.[4,14] It is in most cases discovered some years after the onset of CT infection, in women operated on for tubal infertility or ectopic pregnancy. At this time of the disease, CT detection in the lower genital tract is usually negative, but has been found positive in 5–20% of the male partners' prostatic fluid when detected by culture or PCR after prostatic massage.[26] CT female serology is positive for IgG and IgA without evolutivity on a second serum.[18] Hysterosalpingography and/or laparoscopy show the lesions: hydrosalpinx or incomplete tubal stenosis, pelvic adhesions, often associated with a mild degree of inflammatory reaction, that we called "viscous pelvis"[2,4] lightly red adhesions, too brilliant peritoneal surfaces, viscous and yellow liquid in the cul-de-sac. Tubal and peritoneal histology reveals in most cases some degree of moderate inflammation, with connective tissue oedema, lymphocytes and plasmocytes infiltration as described in France by R. Palmer and J. de Brux[27] and confirmed by other authors.[28]

CT detection is a puzzling and unsolved problem in those chronic cases. We obtained in several assays[29,30] 10–30% positive cultures, and obtained isolates by multiple intra-pelvic sampling and multiple passages. Keilani et al.[31] obtained similar results. CT detection has been found positive in 30% by direct detection, 20% by in situ hybridization on two series of hydrosalpinx biopsies.[11,12] In contrast, most authors published negative results in such cases[32,33] and our own assays of CT detection by PCR on tubal sampling or biopsies remained negative in most TFI cases.[34] These discrepancies are possibly due to the very small number of altered elementary bodies or antigen fragments disseminated in submucosal tissues, as shown by hydrosalpinx biopsies electron microscopy.[12] In vitro studies[10] have shown this persistent form as lacking of protective immunity antigens (MOMP) and having a continuous production of pathogenesis antigens (hsp 60, hsp 70), together with altered antigenic characteristics which may exacerbate the disease process. The antigenic composition of these persistent forms, in addition to their culture-negative characteristics, causes them to be difficult to recognise by either standard cell culture methods or direct antigen detection systems based on the presence of MOMP or LPS. In addition, when the stimulus for persistence is removed, the quiescent organisms resume normal growth, demonstrating that chlamydial viability is maintained during the persistent state.[10]

### Treatment

Is antibiotic treatment recommended in chronic CT infections? In several studies we found antiCT antibiotics inefficient, obtaining intrapelvic positive culture or ISH detection after one to two months of treatment.[12,29] We observed however an improvement of viscous pelvis after a triple antibiotic association: synthetic tetracycline plus ofloxacine for one month, then synthetic tetracycline plus roxithromycin for one month. The interest of this therapeutic sequence has to be confirmed by randomised studies with laparoscopic control.

Reparative microsurgery, mostly performed by laparoscopy to date, continues to be indicated in mild cases. Its success depends on 1) degree of tubal stenosis, incomplete or complete and 2) tubal mucosa damage as objectived by endotuboscopy[35,36] and tubal biopsies. Uterine pregnancy rate after surgery of hydrosalpinges is in the range of 40% if tubal mucosa is normal, less than 20% if it is damaged. IVF is consequently often performed. The influence of CT infection on IVF results also is controversial. A positive antiCT serology or the presence of anti CT HSP antibodies is associated with IVF failure and more particularly to ectopic pregnancies or early spontaneous abortions for certain authors[37,38] but is of no influence for other ones.[39]

Ectopic pregnancy is clinically easy to diagnose, with its bleeding and unilateral pelvic pain associated with early pregnancy signs. Positive beta gonadotrophins in contrast to an empty uterus at sonography are the keys to diagnosis. Laparoscopy is used for both diagnosis and treatment, medical treatment being reserved to very selected cases. Paradoxically, most surgeons are used to correctly treating ectopics, either in a conservative way, by salpingotomy, or more radically, by salpingectomy, but they do not care for evaluation of tubal damage in the tube with ectopic nidation and the contra lateral one, nor to etiological investigations that should be of interest during and after the surgical procedure. If CT infection is involved in a majority of ectopics, this disease can be relevant to other causes, such as endometriosis or a present or past intra uterine device and, in this latter case, has no tendency to recurrence.[40] Further studies, to evaluate the different factors leading to recurrence in women with positive CT serology, are indicated. Positive cultures,[41] positive detection of chlamydial DNA as recently found[42] in 7 of 10 tubes with ectopic pregnancy, and presence in serum of antibody to CT hsp60 are possible candidates to influence reproductive prognosis of a young woman with a first ectopic pregnancy.

### CONCLUSIONS

Considering the cost of PID and late sequelae, together with the doubtful efficiency of their treatments, the best cost/benefit attitude is early diagnosis and treatment of chlamydial infection in the lower genital tract, by systematic screening of young people.

### REFERENCES

1. Mårdh PA, Ripa T, Svenson L, Westrom L: Chlamydia trachomatis infection in patients with acute salpingitis. N Engl J Med 296:1377–1379, 1977.
2. Henry-Suchet J. Catalan F. Loffredo V. et al.: Microbiology of specimens obtained by laparoscopy from controls and from patients with pelvic inflammatory disease or infertility with tubal obstruction. Am J Obst Gynecol Tome 138:1022–1025, 1980.
3. Diquelou JY, Pia P, Tesquier L, Henry-Suchet J, Gicquel JM, Boyer S: La place de *Chlamydia trachomatis* dans l'étiologie infectieuse des grossesses extra-utérines. J Gynecol Obstet Biol Reprod 17:325–332, 1988.
4. Henry-Suchet J, Utzmann C, De Brux J, Ardoin P, Catalan F: Microbiologic study of chronic inflammation associated with tubal factor infertility: Role of *Chlamydia trachomatis*. Fertil Steril 47:274–277, 1987.
5. Schachter J: The Pathogenesis of chlamydial infection. Proc. European Society for Chlamydia Research. Mardh P.A. et al. (eds.) Uppsala. 2:67–72, 1992.
6. Morrison R.P. et al.: Immunology of *Chlamydia trachomatis* infection. In: Quinn TC (ed.) Advances in Host Defense Mechanisms. New York: Raven Press, 1992 pp. 57–64.
7. Witkin SS, Jeremias J, Toth M, Ledger WJ: Cell mediated immune response to the recombinant heat-shock protein of *Chlamydia trachomatis* in women with salpingitis. J Infect Dis 167:1379–1383, 1993.
8. Brunham RC: Vaccine design for the prevention of *Chlamydia trachomatis* infection. Orfila et al. (eds.)Chlamydial Infections. Esculapio, 73–81, 1994.
9. Byrne GI, Arno J, Kane CD: Preliminary caracterisation of local immune responses in women with chlamydial upper genital tract disease. 2nd Proc. Mardh PA et al. (eds).European Society for Chlamydia Research. Uppsala. 88, 1992.
10. Beatty WL, Belinger TA, Le DK, Desai AA, Morrison RP, Byrne GI: Chlamydial persitence mechanism of induction and parrallels to a stress-related response. Orfila J et al. (eds). Chlamydial Infections. Esculapio. 415–418, 1994.
11. Campbell LA, Patton DL, Moore DE, Cappuccio AL, Mueller BA, Wang SP: Detection of *Chlamydia tracho-*

*matis* deoxyribonucleic acid in women with tubal infertility. Fertil Steril 59:45–50, 1993.

12. Patton DL, Askienazy M, Henry-Suchet J, et al: Detection of *Chlamydia trachomatis* in fallopian tube tissue in women with postinfectious tubal infertility. Am J Obstet Gynecol 171:95–101, 1994.
13. Lehtinen M, Paavonen J: Heat-shock proteins in the immunopathogenesis of Chlamydial pelvic inflammatory disease. Orfila J et al. (ed). Chlamydial Infections. Esculapio. pp 599–609, 1994.
14. Boudouris O, Henry-Suchet J, De Brux J, Utzmann C: Infections utéro-annexielles chroniques non spécifiques. Encycl Med Chir Paris Gynecologie 472:1–8, 1989.
15. Henry-Suchet J, Dahan M, Tannous W, Askienazy-Elbhar M: Salpingites aiguës non spécifiques. Conduite à tenir. Editions Techniques-Encycl Méd Chir Paris-Franc 470:1–18, 1995.
16. Wang SP, Eschenbach DA, Holmes KK, Wager G, Grayston JT: *Chlamydia trachomatis* infection in Fitz-Hugh-Curtis syndrome. Am J Obstet Gynecol 138:1034–1038, 1980.
17. Mårdh PA, Lind I, Svensson L, Westrom L, Moller BR: Antibodies to *Chlamydia trachomatis*, *Mycoplasma hominis* and *Neissera gonorrhoeae* in sera from patients with acute salpingitis. Br J Vener Dis 57:125–129, 1981.
18. Henry-Suchet J, Revol C, Askienazy-Elbhar M, Akue BA, Thibon M: Post-therapeutic evolution of serum chlamydial antibody titers in women with acute salpingitis and tubal infertility. Fertil Steril 62:296–304, 1994.
19. Stamm WA, Peeling RW, Money D, et al.: Prevalence and correlates of antibody to Chlamydial HSP-60 in *C. trachomatis* infected women. 8th int. Symposium on Human Chlamydial Inf. proc. 614–617, 1994.
20. Sweet R: Pelvic Inflammatory Disease. Hospital Practice 28:25–30, 1993.
21. Jacobson L: Differential diagnosis of acute pelvic inflammatory disease. Am J Obstet Gynecol 138:1006–1011, 1980.
22. Henry-Suchet J, Soler A, Loffredo V: Laparoscopic treatment of tubo-ovarian abscesses (50 cases). J Reprod Med 29:579–582, 1984.
23. Wolner-Hansen P, Kiviat N, Wasserheit JD, et al.: Etiology of pelvic inflammatory disease: upper genital tract microbiologic studies in patients with confirmed salpingitis and endometritis. Intern Society for STD Research, Brighton, 1985.
24. Westrom L: Reproductive events after acute salpingitis. Brit J Clin Pract 17:17–20, 1982.
25. Westrom L: Consequences of genital Chlamydial infections in women. Proc. European Society for Chlamydia Research. Stary A. (ed). 3:137–140, 1996.
26. Dolivo M, Askienazy-Elbhar M: Intéret du massage prostatique pour la mise en evidence de C.Trachomatis dans l'uretre masculin. Contracept Fertil Steril, 1993.
27. De Brux J, Palmer R: Nomenclature histologique des lésions inflammatoires tubaires. Oviducte et Fertilite - Masson Edit. Paris, 197–204, 1979.
28. Patton DL, Moore DE, Spadoni LR, Soules MR, Halbert SA, Wang SP: A comparison of the fallopian tube's response to overt and silent salpingitis. Obstet Gynecol 73:622–630, 1989.
29. Henry-Suchet J, Douieb P: Treatment of culture documented intrapelvic *Chlamydia trachomatis* infection in women selected for tuboplasties: Preliminary results. Chlamydial Infections New York NY, 201–204, 1986.
30. Askienazy-Elbhar M, Moncan T, Orfila J, Henry-Suchet J, Tannous W, Gautheron C: Cultures de prélevements pelviens dans des chlamydioses aigues et chroniques. Etude des sérotypes obtenus. ISSTDR proceed. Banff Canada, 1991.
31. Keilani A, Boulieu D, Raudrant D, Carraz M, Quenin P: Role de *Chlamydia trachomatis* dans les pathologies tubaires (salpingites aigues et stérilité tubaire). J Gynecol Obstet Biol Reprod 18:167–172, 1989.
32. Mahony J, Sellors J, Chernesky M: Chlamydia western blot analysis of women with laparoscopically confirmed PID and tubal factor infertility (TFI). European Society for Chlamydia Research. Bologne Esculapio. 276, 1988.
33. Bevan C.D. Siddle N.C. Mumtaz G. et al.: *Chlamydia trachomatis* in the genital tract of women with acute salpingitis identified by a qualitative and quantitative PCR before and after treatment. Chlamydial Infections. Orfila J. (ed). Esculapio 350–357, 1994.
34. Thomas, M: Apport de la biologie moleculaire à l'épidemiologie et à la physiopathologie de l'infection chlamydienne. These UTC Compiegne 1:320, 1996.
35. Henry-Suchet J, Veluyre M, Pia P:Etude statistique des facteurs influencant le pronostic des plasties tubaires. J Gynecol Obstet Biol Reprod 18:571–580,1989.
36. Henry-Suchet J, Tesquier L, Pez JP, Loffredo V: Endoscopy of the tube (= tuboscopy): Its pronostic value for tuboplasties. Acta europaea fertilitatis. 16:139–145, 1985.
37. Rowland GF, Forsey T, Moss TR, Steptoe PC, Hewitt J, Darougar S: Failure of in vitro fertilization and embryo replacement following infection with *Chlamydia trachomatis*. J In Vitro Fertiliz Embryo Trans 2:151–155, 1985.
38. Witkin SS, Kligman I, Grifo JA, Rosenwaks Z: *Chlamydia trachomatis* detected by polymerase chain reaction in cervices of culture-negative women correlates with adverse in vitro fertilization outcome. J Infect Dis 171:657–659, 1995.
39. Claman P, Amimi MN, Peeling RW, Toya B, Jessamine P: Does serologic evidence of remote *Chlamydia trachomatis* infection and its heat shock protein (CHSP 60) affect in vitro fertilization-embryo transfer outcome? Fertil Steril 65:146–149, 1996.
40. Job Spira N, Coste J, Aubiel-Cavalier B, et al.: Fréquence de la grossesse extra-utérine et caractéristiques des femmes traitées. Premiers résultats du registre d'Auvergne. Presse Médicale 24:351–355, 1995.
41. Diquelou JY, Pia P, Tesquier L, et al.: Cultures positives pour *Chlamydia trachomatis* chez les femmes ayant une grossesse extra-utérine. Presse Med 16:32, 1987.
42. Gerard HC, Branigan PJ, Minassian SS, Hudson AP: Viability of inapparent *Chlamydia trachomatis* in fallopian tube of ectopic pregnancies. Proc. European Society for Chlamydia Research. A Stary (ed.). 3:296, 1996.
